# IL-1β mediates the induction of immune checkpoint regulators IDO1 and PD-L1 in lung adenocarcinoma cells

**DOI:** 10.1186/s12964-023-01348-1

**Published:** 2023-11-20

**Authors:** Afshan Fathima Nawas, Ashley Solmonson, Boning Gao, Ralph J. DeBerardinis, John D. Minna, Maralice Conacci-Sorrell, Carole R. Mendelson

**Affiliations:** 1 Department of Biochemistry, University of Texas Southwestern Medical Center, 5323 Harry Hines Boulevard, Dallas, TX 75390 USA; 2 Children’s Medical Center Research Institute, University of Texas Southwestern Medical Center, 5323 Harry Hines Boulevard, Dallas, TX 75390 USA; 3 Hamon Center for Therapeutic Oncology Research, University of Texas Southwestern Medical Center, Dallas, TX 75390 USA; 4 Departments of Internal Medicine and Pharmacology, University of Texas Southwestern Medical Center, Dallas, TX 75390 USA; 5 Howard Hughes Medical Institute, University of Texas Southwestern Medical Center, 5323 Harry Hines Boulevard, Dallas, TX 75390 USA; 6 Harold C. Simmons Comprehensive Cancer Center, University of Texas Southwestern Medical Center, Dallas, TX 75390 USA; 7 Department of Cell Biology, University of Texas Southwestern Medical Center, Dallas, TX 75390 USA; 8 Department of Obstetrics and Gynecology and North Texas March of Dimes Birth Defects Center, The University of Texas Southwestern Medical Center, 5323 Harry Hines Boulevard, Dallas, TX 75390 USA

## Abstract

**Introduction:**

Inflammation plays a significant role in various cancers, including lung cancer, where the inflammatory cytokine IL-1β is often elevated in the tumor microenvironment. Patients with lung adenocarcinoma show higher levels of serum IL-1β compared to healthy individual. Moreover, IL-1β blockade reduces the incidence and mortality of lung cancer. Our prior studies revealed that alveolar type-II cells, the precursors for lung adenocarcinoma, display an induction in the expression of the enzyme tryptophan 2,3-dioxygenase (TDO2) during normal lung development. This induction of TDO2 coincides with an increase in IL-1β levels and is likely caused by IL-1β. Given that cancer cells can co-opt developmentally regulated pathways, we hypothesized that IL-1β may exert its pro-tumoral function by stimulating TDO2 and indoleamine 2, 3-dioxygenase-1 (IDO1), parallel enzymes involved in the conversion of tryptophan (Trp) into the immune-suppressive oncometabolite kynurenine (Kyn). Our goal was to determine whether IL-1β is a common upstream regulator of immune checkpoint regulators.

**Methods:**

To determine whether IL-1β regulates IDO1, TDO2, PD-L1, and PD-L2, we measured mRNA and protein levels in lung adenocarcinoma cells lines (A549, H1792, H1838, H2347, H2228, HCC364 and HCC827) grown in 2D or 3D and in immortalized normal lung epithelial cells (HBEC3-KT and HSAEC1-KT). To determine the importance of the NFκB pathway in mediating IL-1β -regulated cellular effects, we used siRNA to knockdown RelA/p65 in IL-1β treated cells. The levels of Trp and Kyn in the IL-1β-treated cells and media were measured by mass spectrometry.

**Results:**

Upon IL-1β stimulation, lung adenocarcinoma cells exhibited significant increases in IDO1 mRNA and protein levels, a response that depended on the NFκB pathway. Interestingly, this induction was more pronounced in 3D spheroid cultures compared to monolayer cultures and was not observed in normal immortalized lung epithelial cells. Furthermore, the conversion of Trp to Kyn increased in cells exposed to IL-1β, aligning with the heightened IDO1 expression. Remarkably, IL-1β also upregulated the expression of programmed death ligand-1 (PD-L1) and PD-L2 in multiple cell lines, indicating that IL-1β triggers parallel immune-suppressive mechanisms in lung adenocarcinoma cells.

**Conclusions:**

Our studies demonstrate that lung adenocarcinoma cells, but not normal immortalized lung epithelial cells, respond to IL-1β signaling by inducing the expression of parallel immune checkpoint proteins that have the potential to promote immune evasion.

Video Abstract

**Supplementary Information:**

The online version contains supplementary material available at 10.1186/s12964-023-01348-1.

## Introduction

Lung cancer is the leading cause of cancer-related deaths in both men and women [1]. Approximately 85% of lung cancers are non-small cell lung cancer (NSCLC), with adenocarcinoma being the main subtype in smokers and non-smokers [2]. Adenocarcinomas are thought to originate from type 2 alveolar epithelial cells through the activation of several oncogenes [3]. Kirsten rat sarcoma viral oncogene homolog (KRAS) and epidermal growth factor receptor (EGFR) are the most frequently mutated oncogenes in human lung adenocarcinoma. Mutations in liver kinase B1 (LKB1), v-raf murine sarcoma viral oncogene homolog B (BRAF) and anaplastic lymphoma kinase (ALK) rearrangement are less common. These driver mutations dictate which targeted therapy would be the most effective [4–6].

T- cells possess the ability to detect and eliminate cancer cells, but cancer cells can develop strategies to evade this immune response by activating immune-evading pathways. This underscores the significance of employing immune checkpoint blockade as a treatment approach for non-small cell lung cancers (NSCLCs) lacking oncogenic driver mutations like EGFR, BRAF, or EML4-ALK, which can be specifically targeted therapeutically [7]. However, immune checkpoint blockade therapy provides long-term benefits to only a minority of NSCLC patients, typically ranging from 15 to 20% [7]. A significant knowledge gap exists regarding the mechanisms employed by NSCLCs to evade immune surveillance during their development, as well as the factors determining why some NSCLCs respond to immune checkpoint blockade while others do not.

Two crucial pathways in cancer immune evasion are: the increased expression of programmed cell death ligand-1 (PD-L1), and the upregulation of the Trp metabolite, Kyn [8, 9]. PD-L1, present on the surface of cancer cells, interacts with the programmed cell death protein-1 (PD-1) on the surface of T-cell, resulting in T-cell inactivation and promoting tumor progression [6, 10]. Kyn present within the tumors can lead to suppression of cytotoxic T-cell activation and result in a pro-tumor microenvironment [11, 12]. TDO2 and IDO1 are the rate limiting enzymes necessary for the conversion of Trp to Kyn [13]. IDO1 expression is elevated in lung adenocarcinomas relative to the normal adjacent tissue and correlates with advanced clinical stage and lymph node metastasis [14]. Relative expression of IDO1 mRNA levels were shown to be higher in lung tumor samples relative to lung cancer cell lines, indicating that factors within the tumor microenvironment could induce IDO1 expression [15]. TDO2 expression is also elevated in lung adenocarcinomas compared to normal tissue [16]. A co-culture model showed that lung cancer-derived galectin-1 induced TDO2 expression in cancer associated fibroblasts [17].

The inflammatory cytokine interleukin-1β (IL-1β) is commonly elevated in tumors from various origins, such as lung, breast, prostate, colon, and head and neck cancers, and its elevated levels are associated with unfavorable patient outcomes [18–21]. In NSCLC patients, IL-1β levels are higher in tumor tissues than in adjacent normal tissue [20]. Serum IL-1β levels in lung cancer patients are notably elevated compared to those in healthy individuals, and these heightened levels are also associated with worse clinical outcomes [22]. Importantly, a retrospective analysis of the Canakinumab Anti-inflammatory Thrombosis Outcomes Study (CANTOS) showed that treatment with anti-IL-1β antibody, canakinumab, drastically reduced incidence and mortality of lung cancer in patients with prior myocardial infraction [23]. However, the mechanism underlying this clinical benefit remains unclear. We recently reported that TDO2, but not IDO1, is upregulated in human fetal alveolar epithelial cells and in the mouse fetal lungs by the transcription factor nuclear factor erythroid 2-related factor 2 (NRF2) [24], which occurs concomitantly with an upregulation of IL-1β [25].

Given that cytokines have the capacity to stimulate the expression of enzymes such as IDO1 and TDO2, and taking into account that lung adenocarcinoma is thought to originate from type-II alveolar epithelial cells that are capable of producing IL-1β [26, 27], we hypothesized that IL-1β stimulates enzymes that facilitate Kyn production in lung adenocarcinoma cells. Our study shows that IDO1 and IDO1’s product Kyn are elevated in lung cancer cells exposed to IL-1β. Moreover, we found that IL-1β exposure also elevated PDL-1 levels in lung cancer cells. Our study defines a mechanism that allows cancer cells to evade T-cell activity via two parallel and potentially redundant mechanisms.

## Materials and methods

### Cell culture

Lung cancer cell lines, NCI-H1792, NCI-H1838, NCI-H2228, NCI-H2347, HCC364 and HCC827 and normal cells: human bronchial epithelial cells (HBEC3-KT) and human small airway epithelial cells (HSAEC1-KT) both immortalized with cyclin-dependent kinase 4 (CDK4) and human telomerase reverse transcriptase (hTERT) were obtained from the Hamon Center Repository [28, 29]. HBEC3-KT and HSAEC1-KT were maintained in keratinocyte serum free medium (KSFM). A549 cell line was purchased from ATCC. All cell lines were DNA fingerprinted (PowerPlex Fusion Kit, Promega) for provenance and found to be mycoplasma free (Myco Kit, Boca Scientific). Lung cancer cell lines were maintained in RPMI-1640 (Fisher; 11–875-135) supplemented with 5% FBS (Gibco; 10437–028). HCC827 and H1792 cells were used for spheroid culture in low attachment plates/dishes. Using 1% agarose-coated 96-well plates, 1 × 10^4^ cells were plated per well in RPMI supplemented with 5% FBS. Spheroids formed spontaneously between 24 to 48 h and were collected for analysis on day 5 after seeding. To isolate protein and RNA, the cells were plated on 1% agarose-coated 6- or 10-cm dishes and collected after the same duration.

### RNA extraction and RT-qPCR

Total RNA from cells was extracted using RNeasy Mini Kit (Qiagen; 74004). RNA was incubated with DNase (Invitrogen; 18068015) to remove any contaminating DNA, and 1 μg of RNA was reverse transcribed using the iScript Reverse Transcription Supermix (Bio‐Rad; 170–8841). For quantitative analysis of mRNA, a BioRad CFX384 Real‐Time PCR Detection System was used with iTaq Universal SYBR Green Supermix (Bio‐Rad; 172–5125) for the detection of PCR products. The cycling conditions were 95 °C for 30 s, followed by 39 cycles of 95 °C for 15 s, and 60 °C for 30 s. Each sample was analyzed in triplicate, and the mean of Cq value was calculated. The relative fold changes were calculated using the comparative Ct method (2–ΔΔCt). Primers used in RT‐qPCR are listed in Table S1. mRNA levels were normalized to 18s mRNA.

### Western blotting

Western blot analysis was done as previously described [30]. Briefly, cells were lysed in NP40 lysis buffer (0.5% NP40, 50 mM of Tris [pH 7.5], 150 mM of NaCl, 3 mM of MgCl_2_, 1X protease inhibitor, 1X phosphatase inhibitor). Protein concentration was measured using the Pierce BCA Protein Assay Kit (Thermo Fisher Scientific; 23,227). For Western blot analysis, 20–30 µg protein was separated using a 10% sodium dodecyl sulfate polyacrylamide gel (Invitrogen, NP0316BOX). Proteins were transferred from the gel to 0.45 μm pore size nitrocellulose membrane and total protein visualized using Ponceau S. The membrane was blocked with 2.5% (wt/vol) BSA (Thermo Fisher Scientific; BP 1600–1) in 1X tris-buffered saline with Tween 20 (TBST; 20 mM of Tris, pH 7.6, 150 mM of NaCl, 0.05% Tween-20). Primary and secondary antibodies were diluted in 2.5% BSA in 1X TBST. Protein blot bands were visualized using SuperSignal West Femto Maximum Sensitivity Substrate (Thermo Fisher Scientific; 34,095). Primary antibodies included IDO1 (Cell signaling; 86630S), RelA/p65 (Cell Signaling; 6956S), PD-L1 (Cell signaling; 13684 T), GAPDH (Cell Signaling; 5174 T) and the secondary antibody was Peroxidase-linked anti-Rabbit IgG (Fisher; 45–000-683).

### LC–MS/MS Quantification of L-Trp and L-Kyn

Cells were incubated in their regular growth media (RPMI-1640, 5% FBS) with vehicle control (PBS) or 5 ng/mL IL-1β for the denoted duration. After incubation, 500 µL of the media were collected, centrifuged at 14,000 rpm for 5 min to pellet cells and other debris. The supernatants were snap frozen in liquid nitrogen and stored at -80ºC until further processing. The cells were washed twice with 0.9% NaCl (Baxter) to remove any residual media and then collected in 80:20 acetonitrile:water (Optima LCMS grade) and flash frozen in liquid nitrogen and stored at -80ºC until further processing. Polar metabolites were extracted from cells by 3 rounds of freeze–thaw in liquid nitrogen and 37ºC water bath to lyse cells. Aliquots (50 µL) of the medium were added to 950 µL 80:20 acetonitrile:water (Optima LCMS grade). Samples were centrifuged at 14,000 rpm for 10 min and supernatant was transferred to a fresh tube. Extracts were subjected to BCA assay (Pierce) and normalized to 7 µg in 100 µL of 80:20 acetonitrile before LCMS analysis. Metabolite analysis used a Vanquish UHPLC coupled to a Thermo Scientific QExactive HF-X hybrid quadrupole orbitrap high-resolution mass spectrometer (HRMS) as performed previously [31, 32]. Relative metabolite abundance was determined by integrating the chromatographic peak area of the precursor ion searched within a 5-ppm tolerance and then normalized to total ion count (TIC).

### Immunofluorescence

Immunofluorescence was performed as previously described [30]. Spheroids were transferred to a fresh multi-well plate and fixed with 4% paraformaldehyde for 60 min at room temperature then permeabilized with 0.25% Triton in PBS for 60 min. Fixed spheroids were blocked with 2.5% BSA in 1 × PBS at room temperature for at least 60 min. Antibodies were diluted in 2.5% BSA in 1 × PBS. Spheroids were incubated in primary antibody overnight at 4 °C, washed with 1 × PBS, and then incubated with fluorescently labeled secondary antibody overnight at 4 °C in the dark. Fluorescently labeled secondary antibody: Alexa Fluor 488, goat anti-mouse (Invitrogen, Waltham, MA; A11001). Nuclei were stained with DAPI. Subsequently, the spheroids were cleared with 88% glycerol in PBS for at least 1 h at room temperature and transferred to a glass bottom 96- or 24-well plate (MatTek Corp., P24G-1.5–10-F) and then imaged at 20X magnification using a Zeiss LSM880 with Airyscan microscope. To quantify the percentage of IDO1^+^ cells, first the DAPI stained blue nuclei were counted to obtain total cell number and the red fluorescent cells were counted to obtain number of IDO1^+^ cells. Results are represented as % IDO1 + cell/total cells in an entire section of the spheroid.

### Cell viability analyses

Cell viability assay was performed as previously described [33–35]. For monolayer culture, viable cells were fixed with cold 100% methanol for 15 min and washed twice with 1 × PBS. The nuclei were then stained with DAPI dissolved in 1 × PBS. The nuclei were imaged on the Cytation5 Imaging Reader and counted using the Gen5 Microplate Reader and Imager Software (Agilent/BioTek). 1 nucleus was counted as 1 cell. For spheroids, CellTiterGlo Assay (G9681; Promega) was performed. 1 × 10^4^ HCC827 or H1792 cells were plated per well in 1% agarose-coated 96-well plates. Spheroids formed spontaneously in 24–48 h and CellTiter Glo assay was performed according to manufacturer’s instructions on day 4 after seeding. The spheroids and 100 µL of the media were transferred to a White opaque 96-well microplate (PerkinElmer; 6,005,680) and 50 µL of the CellTiter Glo 3D reagent was added, covered in aluminum foil and mixed by shaking for 5 min. The plate was then incubated for another 25 min at room temperature and the luminescence was measured using Cytation5 Imaging Reader.

### TCGA data analysis

Data containing the mRNA expression of 503 patients with NSCLC adenocarcinoma (TCGA, PanCancer Atlas) was retrieved from The Cancer Genome Atlas using the cBioPortal (http://www.cbioportal.org) [36]. We analyzed the mRNA expression of *IDO1*, *CD274* (*PD-L1*) and *PDCD1LG2* (*PD-L2*) genes. Co-expression data was plotted on GraphPad Prism.

### siRNA transfection

Cells were transfected with siGENOME human *RELA* (p65) siRNA (Horizon; T-2001–02) using DharmaFECT 1 transfection reagent (Horizon; T-2001–02). Non-targeting siRNA duplex was used as a negative control (Horizon; D-001210–02-20). Knockdown was performed using 40 nM siRNA treatment 6 h before IL-1β treatment. RT-qPCR was used to confirm mRNA knockdown.

### Statistical analysis

All experiments were repeated a minimum of 3 times. Statistical significance was determined using unpaired student t-test. *P*-value ≤ 0.05 was considered statistically significant. Graphs are shown as the average of a minimum of 3 biological replicates ± standard deviation (SD) and plotted using Prism Graphpad. All data points are shown on the graphs.

## Results

### IL-1β induces IDO1 expression in lung adenocarcinoma cells but not in normal lung epithelial cells

**Fig. 1:**
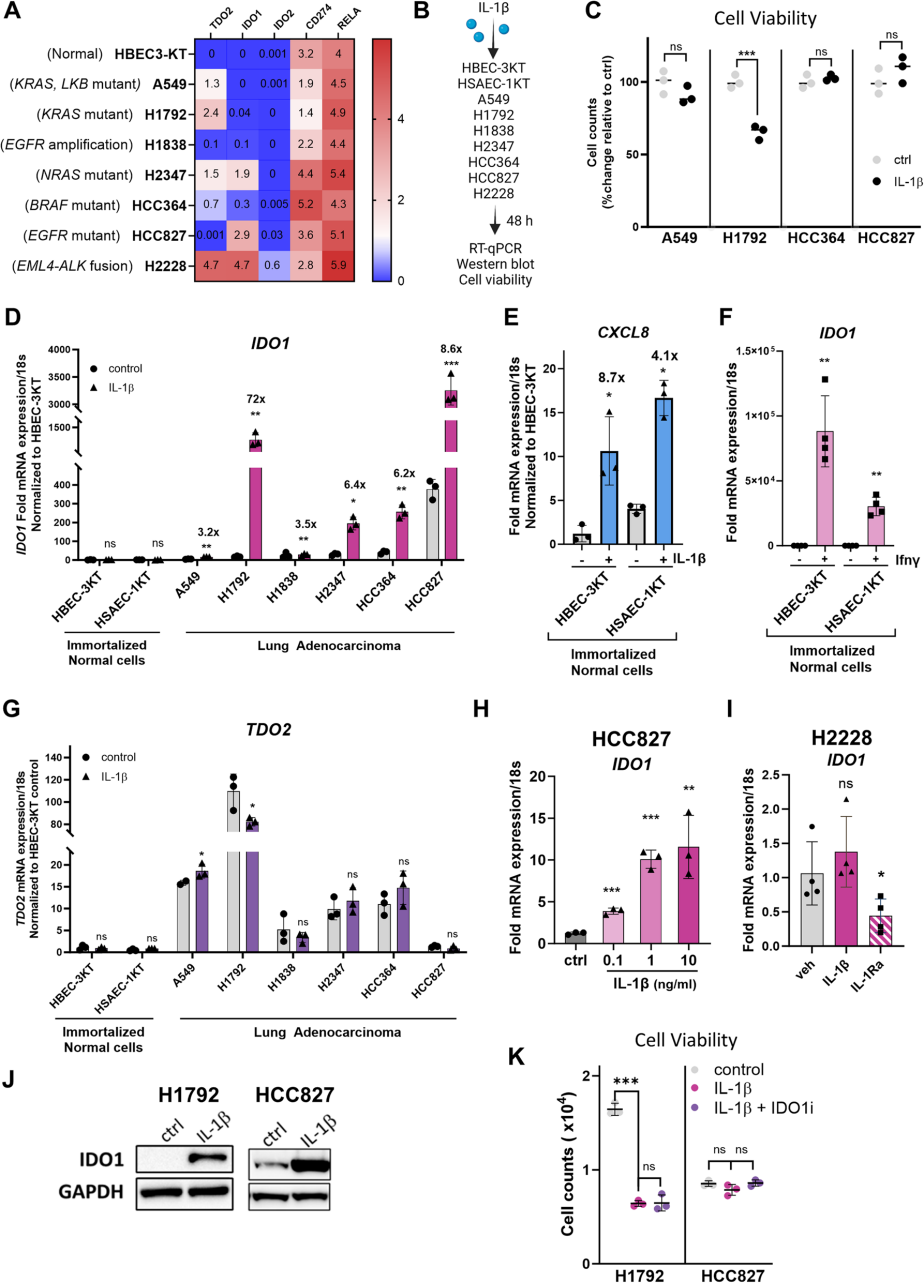
Induction of IDO1 mRNA and protein levels in lung adenocarcinoma cell lines stimulated with IL-1β. **A** Heatmap showing the FPKM values of *TDO2, IDO1, IDO2, CD274* and *RelA* mRNA of the cell lines used in this study. **B** Schematic of experimental set-up illustrating the cell lines tested.** C** Lung adenocarcinoma cells were treated with 5 ng/mL IL-1β, and cell viability measured 48 h after stimulation (*n* = 3 biological replicates). **D** HBEC-3KT and HSAEC-1KT “normal” lung epithelial cells or lung adenocarcinoma cell lines were grown in medium with vehicle control or 5 ng/mL IL-1β for 48 h, and RT-qPCR of *IDO1* was performed. Note split scale for IDO1. **E** RT-qPCR showing induction of *CXCL8* mRNA as a measure of activation of IL-1 signaling in HBEC-3KT and HSAEC-1KT cells in response to 5 ng/ml IL-1β treatment for 48 h.** F** HBEC-3KT and HSAEC-1KT cells show an induction of *IDO1* mRNA in response to 5 ng/mL Ifnγ treatment for 48 h.** G** *TDO2* mRNA levels measured by RT-qPCR in normal and lung adenocarcinoma cells in response to IL-1β treatment for 48 h.** H** IDO1 mRNA levels were measured by RT-qPCR in HCC827 cells treated with 0.1, 1, or 10 ng/mL of IL-1β for 48 h.** I** RT-qPCR of IDO1 mRNA in H2228 cells treated with 5 ng/mL IL-1β or 100 ng/mL IL-1Ra for 48 h. **J** Western blot analysis was performed for IDO1 and TDO2 protein levels in H1792 and HCC827 lung adenocarcinoma cells treated with vehicle control or 5 ng/mL IL-1β for 48 h. GAPDH was used as the loading control. **K** Cell counts were measured for monolayer cultures of H1792 and HCC827 cells stimulated with 5 ng/mL IL-1β ± 20 nM epacadostat (IDOi) for 4 days. Error bars represent ± SD of 3 biological replicates; *P ≤ 0.05, ** ≤ 0.005, *** ≤ 0.0005. mRNA levels were normalized to 18s mRNA and represented as fold change over HBEC-3KT control treatment. GAPDH was used as the loading control

Since TDO2 was previously found to be upregulated concomitantly with IL-1β in normal lung cells [25], we asked whether IL-1β had the ability to regulate Trp-metabolizing enzymes in lung cancer cells. We chose 7 lung cancer cell lines based on the variable levels of expression of IDO1 and TDO2 (Fig. 1A). These cell lines harbor several different oncogenes representing a broad mutation profile that was observed in lung adenocarcinoma patients (described in Table 1). RNAseq data for the 7 lung cancer cell lines (Minna lab, dbGaP Study Accession phs001823.v1.p1), we determined that *TDO2* mRNA was elevated in A549, H1792, H2347, and H2228 cells, while IDO1 and IDO2 were low in most cell lines (Fig. 1A). The normal immortalized lung epithelial cells HBEC-3KT did not express TDO2, IDO1, or IDO2 (Fig. 1A). We incubated HBEC-3KT and HSAEC-1KT, as well as the 7 lung adenocarcinoma cells of varying driver mutations (Table 1) with 5 ng/mL IL-1β for 48 h and performed RT-qPCR, Western blotting, and cell viability assays to determine their response to IL-1β (Fig. 1B). We found that IL-1β did not induce proliferation of the A549, H1792, HCC364 and HCC827 cell lines (Fig. 1C). In agreement with previous studies in breast and prostate cancers [34, 35, 37, 38], these results indicate that IL-1β does not promote cell-autonomous growth advantages to lung adenocarcinoma cells in vitro.

**Table 1:** Lung adenocarcinoma cells and driver mutation

	**Cell line** **(Male/ female)**	**Mutation**	***IL1R1*** **mRNA**	***IL1Racp*** **mRNA**	***IDO1* Response to IL-1β**	***CXCL8* response to IL-1β**
NSCLC Adenocarcinoma	A549 (M)	KRAS, LKB mutant	** + + + + **	** + + **	** + + **	** + + + **
	H1792 (M)	KRAS mutant	** + + + + **	** + + + **	** + + + + + + **	** + + + + + + **
	H1838 (F)	EGFR Amplification	** + + + **	** + + + **	** + + **	** + + **
	H2347 (F)	NRAS mutant	** + + + **	** + + + **	** + + + **	** + + + + **
	HCC364 (M)	BRAF^V600E^ mutant	** + + + + + **	** + + + **	** + + + **	** + + + + **
	HCC827 (F)	EGFR mutant	** + + + **	** + + + + **	** + + + + **	** + + **
	H2228 (F)	EML4-ALK1 fusion	** + + + **	** + + + + + + **	**-**	** + + + **
Immortalized Normal cells	HBEC-3KT	Non-cancerous lung epithelial cells Immortalized with CDK4 and hTERT	** + **	** + + + + **	**-**	** + **
	HSAEC-1KT	Non-cancerous lung epithelial cells Immortalized with CDK4 and hTERT			**-**	** + **

We then asked whether IL-1β activates pathways that lead to immune tolerance of lung cancer cells. We observed a significant upregulation of IDO1 mRNA (Fig. 1D) in A549, H1792, H1838, H2347, HCC364 and HCC827 adenocarcinoma cell lines incubated with IL-1β. Both HBEC-3KT and HSAEC-1KT cell lines displayed low expression of IDO1, and no additional induction was observed with IL-1β stimulation (Fig. 1D). Despite the lack of IDO1 induction, upon incubation with IL-1β, HBEC-3KT and HSAEC-1KT cells induced the expression of *CXCL8* mRNA, a downstream target of IL-1 signaling [34, 39], indicating that these normal cells have the IL-1 receptors as well as an intact downstream signaling cascade in response to IL-1β stimulation (Fig. 1E). In addition, the mRNA expression of IL-1 receptors, *IL-1R1* and *IL-1RAcp* (Minna lab dataset, dbGaP Study Accession is phs001823.v1.p1), and response to IL-1β stimulation, which was measured as fold change in *CXCL8* induction, were detected in immortalized normal cells and lung adenocarcinoma cells (Table 1 and Supplemental Fig. 1A). To determine whether the *IDO1* gene and promoter are intact in normal cells, we treated the cells with a known potent inducer, interferon gamma (Ifnγ), and found that Ifnγ could induce *IDO1* mRNA (Fig. 1F). This indicates that normal cells can respond to cytokine stimuli to induce IDO1, but the IL-1β-mediated induction of IDO1 was specific to cancer cells.

Since both IDO1 and TDO2 can catalyze the conversion of Trp to Kyn, we asked whether IL-1β can also induce TDO2 expression in adenocarcinoma cells. Both HBEC-3KT and HSAEC-1KT cell lines had low TDO2 expression and showed no induction with IL-1β (Fig. 1G). Although A549 and H1792 cell lines had high expression of TDO2, none of the lung adenocarcinoma cells showed a significant induction with IL-1β stimulation (Fig. 1G). These results suggest that IL-1β specifically regulates *IDO1* expression in lung adenocarcinoma cells.

*IDO1* expression was IL-1β dose-dependent in the HCC827 cell line, with maximal expression observed at 1 ng/mL IL-1β (Fig. 1H). Interestingly, no further IDO1 mRNA induction with IL-1β occurred in H2228 cells, which had the highest IDO1 mRNA levels, showed (Fig. 1I). Surprisingly, the cancer cell line encyclopedia (CCLE) dataset revealed that H2228 cells also expressed elevated mRNA levels of *IL-1α* and *CXCL8* (downstream target of IL-1 signaling) (Supplemental Fig. 1B). Given that IL-1α also induced similar signaling cascade as IL-1β, we next asked whether autocrine signaling by IL-1α in H2228 cells is responsible for elevated IDO1 mRNA levels in these cells. We incubated H2228 cells with IL-1 receptor antagonist (IL-1Ra), a natural antagonist to IL-1 signaling, and observed a decrease in IDO1 mRNA (Fig. 1I), indicating that autocrine signaling through IL-1α could be contributing to the elevated IDO1 levels in these cells.

A significant increase in IDO1 protein was observed in H1792 and HCC827 cells treated with IL-1β (Fig. 1J). We tested the effect of the potent inhibitor of IDO1 activity (IDOi) epacadostat [40] on monolayer cultures of H1792 and HCC827 cells treated with IL-1β. Cell counts revealed that epacadostat at 20 nM concentration for 4 days did not decrease viability of these cells (Fig. 1K), indicating that IDO1 activity may not directly induce lung cancer cell growth in monolayer cultures. Our data indicate that regardless of the driver mutations, lung adenocarcinoma cells induce immunosuppressive IDO1 mRNA and protein, but not TDO2, in response to IL-1β stimulation.

### Culturing lung cancer cells in 3D enhances the induction of IDO1 mRNA and protein by IL-1β

**Fig. 2:**
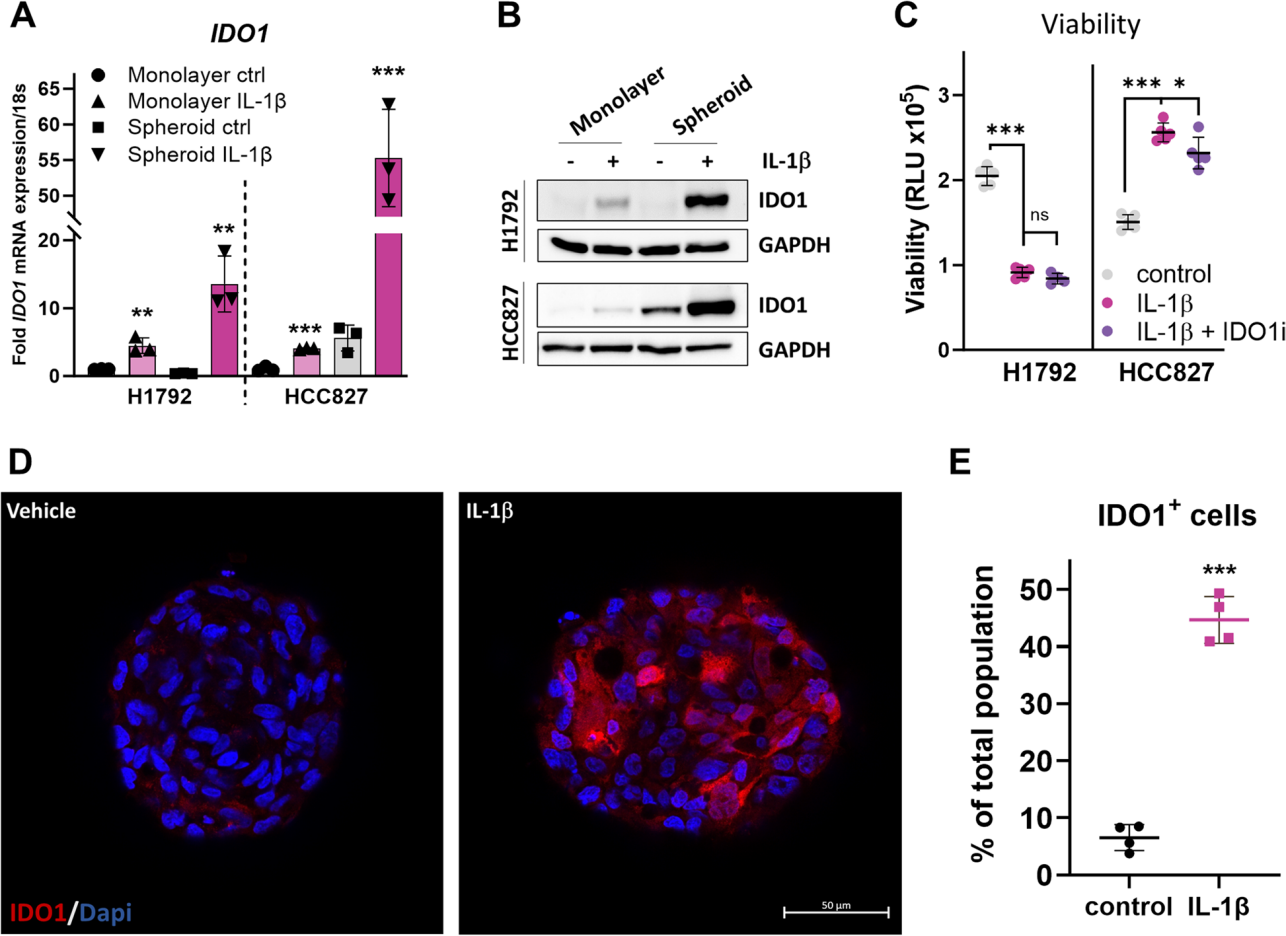
Regulation of IL-1β-mediated IDO1 induction in 3D spheroid model of lung adenocarcinoma cells. **A** RT-qPCR measurement of *IDO1* mRNA in H1792 and HCC827 monolayer cells and spheroids generated in 1% low attachment dishes, in control or IL-1β containing medium for 5 days. mRNA levels were normalized to 18s mRNA and represented as fold change over control treatment. **B** Western blot for IDO1 protein in H1792 and HCC827 monolayer and spheroid cultures ± 5 ng/mL IL-1β for 5 days. GAPDH was used as the loading control. **C** Viability of H1792 and HCC827 spheroids stimulated with 5 ng/mL IL-1β ± 20 nM Epacadostat (IDOi) for 5 days was measured with CellTiterGlo 3D assay (*n* = 4 spheroids). Luminescence was measured and viability is represented as relative luminescence units (RLU). **D** IL-1β induces IDO1 expression within the cells of lung adenocarcinoma spheroids. HCC827 spheroids were grown in low attachment conditions in vehicle control or 5 ng/ml IL-1β for 5 days were immunostained for IDO1 (Texas red, red) and nucleus (DAPI, blue). **E** IL-1β increases the percentage of IDO1^+^ cells in the spheroid. The number of IDO1 + cells were counted and represented as a percentage of the total cell population for *n* = 4 images. Each image of spheroid counted contained between 60—185 total cells. Error bars represent ± SD of 3 biological replicates; *P ≤ 0.05, ** ≤ 0.005, *** ≤ 0.0005

To account for complexity of cell-to-cell contacts and formation of a hypoxic core formed under 3D growth conditions, we tested whether IL-1β–mediated induction of IDO1 in lung adenocarcinoma cells was conserved in a spheroid model of cell culture. We chose to study HCC827 and H1792 cell lines since they had the highest induction of IDO1 among the lung cancer cell lines studied (Fig. 1D) and they form spontaneous spheroids in low attachment conditions. Spheroids were formed in low attachment culture conditions in medium with or without 5 ng/mL IL-1β for 5 days. Given the spheroids take about 48 h to form, they were incubated for an additional 72 h before measuring the changes in IDO1 expression. Monolayer cultures were established for the same duration and conditions in parallel. Both cell lines displayed a more dramatic induction of IDO1 mRNA (Fig. 2A) and protein (Fig. 2B) when grown as spheroids compared to monolayer cultures, possibly due to the altered properties of the cells cultured in the 3D shape. Intriguingly, HCC827 cells appear to have higher IDO1 mRNA and protein in spheroid culture than in the monolayer cultures, which was also reported in CFPAC-1 pancreatic adenocarcinoma cells [41]. Similar induction was also observed in triple-negative breast cancer cells, but intriguingly, only TDO2, was basally higher in cells cultured under low attachment conditions than in monolayer cultures [42], indicating a cell-type or cancer-type specificity.

We next tested whether inhibiting IDO1 activity could suppress spheroid growth of lung adenocarcinoma cells. Surprisingly, IL-1β induced spheroid growth of HCC827 cells, but not H1792 cells, and this increased spheroid growth was modestly inhibited by epacadostat (Fig. 2C). Further, immunofluorescence revealed that a subset of HCC827 cells had elevated IDO1 protein when exposed to IL-1β (Fig. 2D). IDO1^+^ cells were present throughout the spheroid. About 42% IDO1^+^ cells were present within an IL-1β–treated spheroid and 8% in control-treated cells (Fig. 2E). Our data indicates that IL-1β-mediated induction of IDO1 is conserved in spheroid model of cell culture.

### IL-1β regulates *IDO1* transcription by activating the NFκB pathway in lung adenocarcinoma

**Fig. 3:**
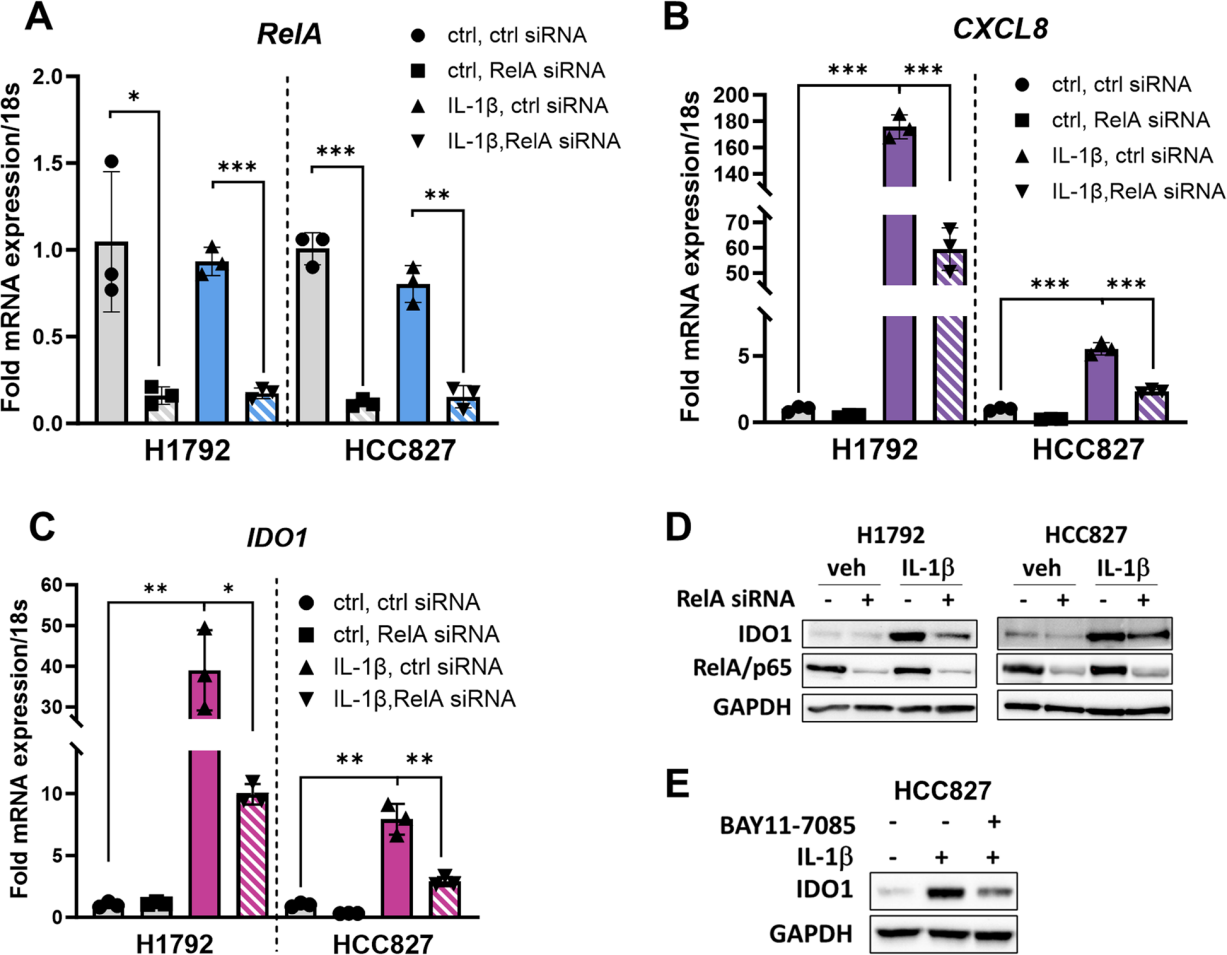
IL-1β regulates IDO1 transcription by activating the NFκB pathway in lung adenocarcinoma. **A** H1792 and HCC827 cells transfected with 40 nM of non-targeting or *RELA*/p65 siRNA (Dharmacon) for 24 h followed by treatment with vehicle (ctrl) or 5 ng/mL IL-1β for 48 h and RT-qPCR for *RELA* mRNA was performed to validate the efficacy of knockdown. **B** RT-qPCR for *CXCL8* mRNA indicates repression of p65 transcriptional activity in H1792 and HCC827 cells. **C** RT-qPCR for *IDO1* mRNA levels indicates p65 regulates *IDO1* transcription in H1792 and HCC827 cells. **D** Western blot showing levels of IDO1 and p65/RelA protein levels. **E** Western blot of HCC827 cells treated with 5 ng/ml IL-1β ± BAY11-7085 (p65/NFκB inhibitor) for 48 h. Error bars represent ± SD of 3 biological replicates: *P ≤ 0.05, ** ≤ 0.005, *** ≤ 0.0005. mRNA levels were normalized to 18s mRNA and represented as fold change over control treatment. GAPDH is the Western blot loading control

An early event in IL-1 signaling is the activation of p65 (*RelA* gene) [43]. To determine whether p65 mediates IL-1β induction of IDO1, we silenced *RelA* mRNA in H1792 and HCC827 cells and then treated with vehicle or IL-1β (5 ng/mL) for 48 h. RT-qPCR and Western blotting showed that *RelA* mRNA (Fig. 3A) was effectively knocked down. To assess whether transcriptional activity of p65 was repressed, we measured the mRNA expression of *CXCL8,* a known gene target of the p65 transcription factor. *CXCL8* mRNA (Fig. 3B) was also downregulated in both cell lines, indicating that p65 transcriptional activity was also reduced. We then analyzed the effect of p65 knockdown on *IDO1* mRNA levels. *RelA* knockdown repressed the IL-1β mediated mRNA (Fig. 3C) and protein induction (Fig. 3D) in H1792 and HCC827 cells. Additionally, we used BAY-17085, an inhibitor of p65 nuclear translocation, to further validate the role of p65 in inducing IDO1 in adenocarcinoma cells (Fig. 3E). Our results demonstrated that the NFκB transcription factor p65 regulated IDO1 in IL-1β-stimulated cells.

### IL-1β induces enzymatically active IDO1 protein

**Fig. 4:**
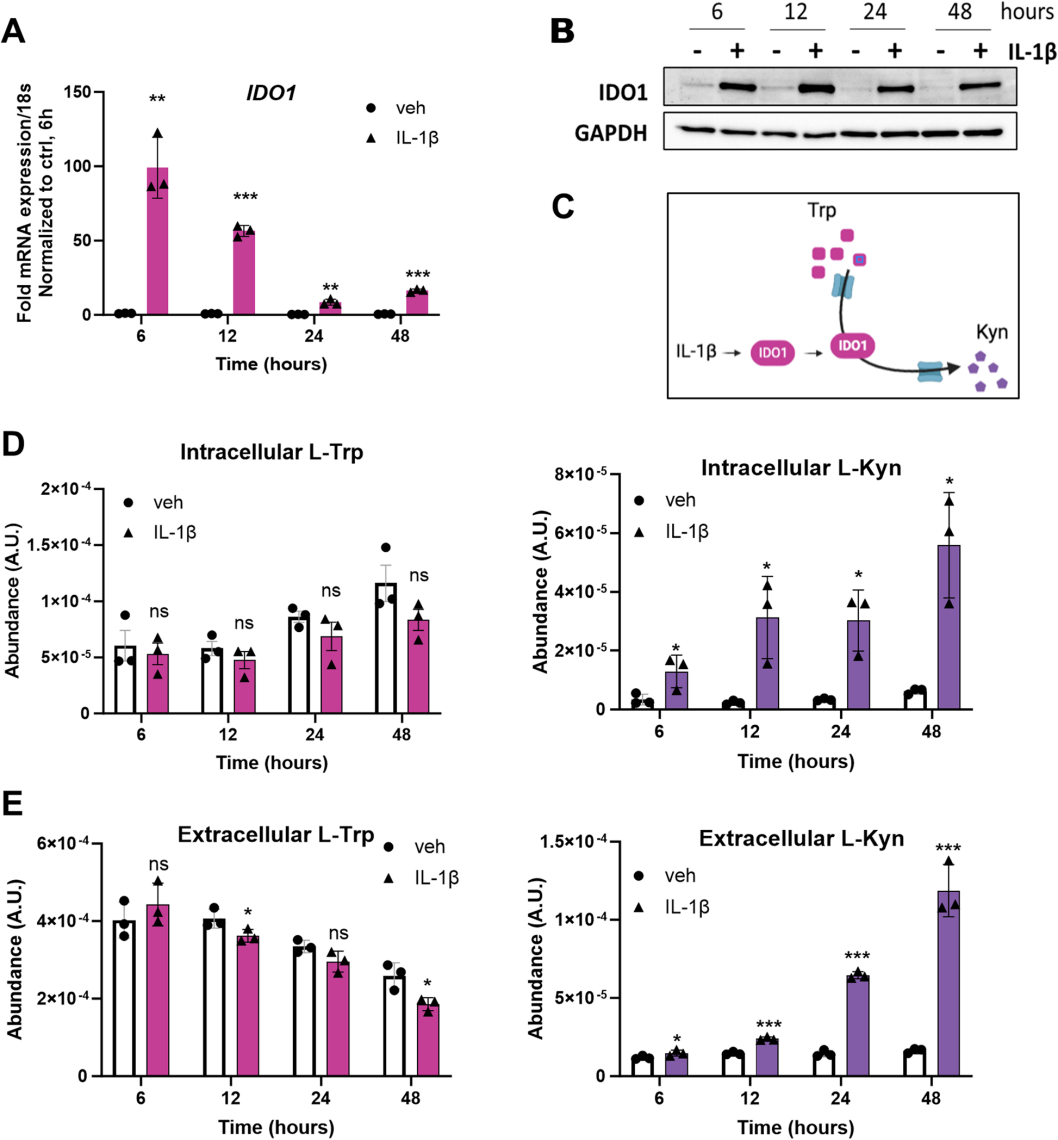
Measurement of IDO1 activity. **A** Level of *IDO1* mRNA induction over time was measured by RT-qPCR of HCC827 cells grown in media with vehicle control or 5 ng/ml IL-1β for 6, 12, 24 or 48 h. mRNA levels were normalized to 18s mRNA and represented as fold change over control at 6 h. **B** Accumulation of IDO1 protein over time is shown by western blotting. GAPDH was used as the loading control. **C** Schematic of the Trp/Kyn pathway. **D** – **E** Intracellular (**D**) and extracellular (**E**) L-Trp and L-Kyn levels were measured as an indicator of IDO1 enzymatic activity. Relative metabolite content is normalized to total ion count. Error bars represent ± SD of 3 biological replicates: *P ≤ 0.05, ** ≤ 0.005, *** ≤ 0.0005

To quantify the enzymatic activity of IDO1 in IL-1β–treated cells, we first determined the kinetics of IDO1 induction by IL-1β using HCC827 cells. *IDO1* mRNA and protein induction was maximal at 6 h after treatment, and the levels decreased until 24 h and then stayed constant until 48 h (Fig. 4A, B). We measured the intracellular and extracellular Kyn levels at 6, 12, 24 and 48 h using LC–MS. Mass spectrometry analysis revealed that intracellular Kyn levels increased significantly in the cells stimulated with IL-1β relative to the control by 6 h post treatment and a continued increase up to 48 h later (Fig. 4D); intracellular Trp levels remained unchanged. Extracellular Kyn levels were also detected in the medium by 6 h post IL-1β treatment relative to the control, and continued to increase to 48 h post treatment (Fig. 4E) and the extracellular Trp levels were significantly lower at 12 and 48 h. These data show that enzymatically active IDO1 protein is induced by IL-1β in lung adenocarcinoma cells.

### IL-1β is a common upstream regulator of immune checkpoint proteins

**Fig. 5:**
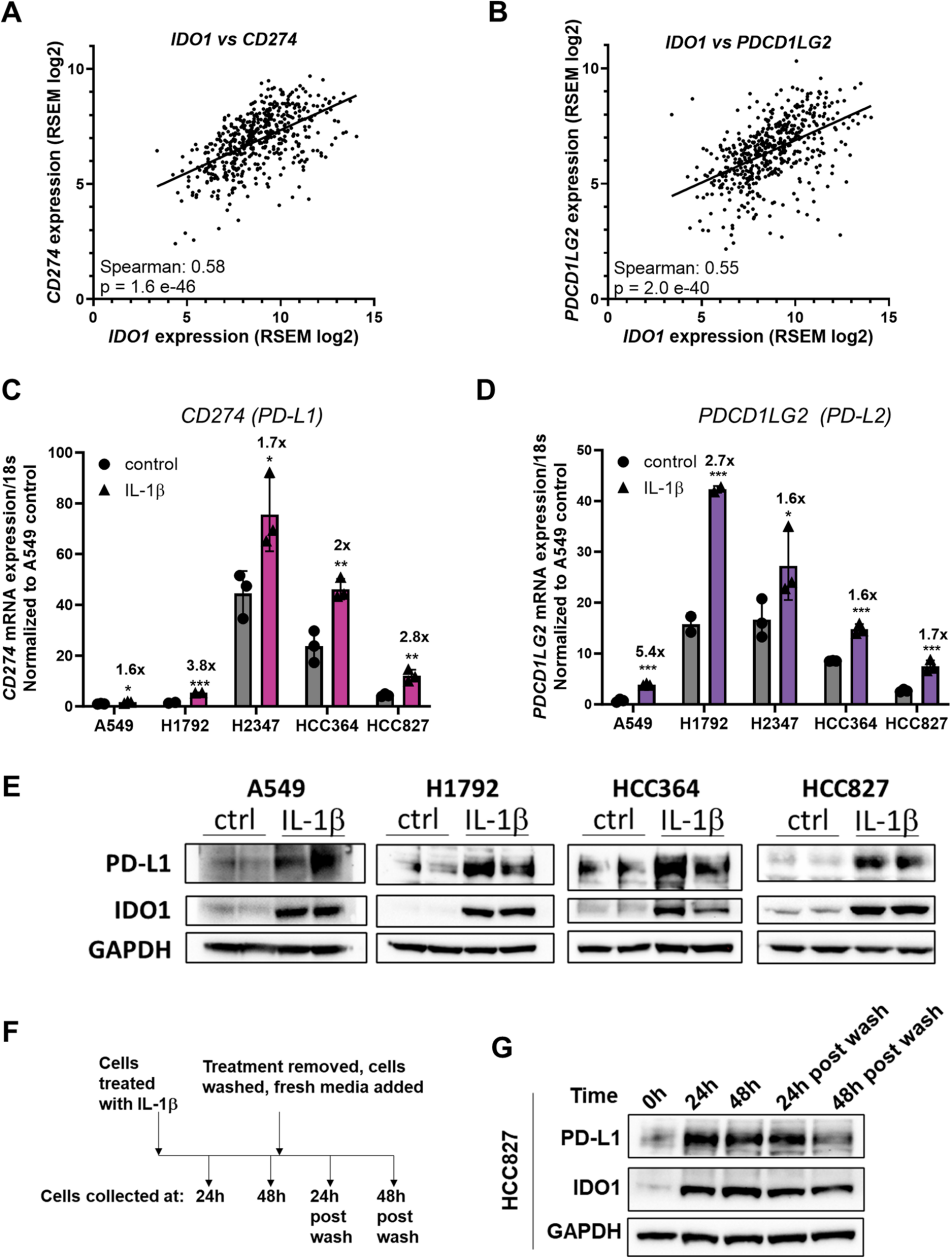
IL-1β mediates PD-L1 and PD-L2 induction concomitant with IDO1 induction in lung adenocarcinoma cells. **A** Expression of *CD274* and *IDO1* mRNA in human lung adenocarcinoma. Each dot represents a lung adenocarcinoma tumor from TCGA (*n* = 510). **B** Expression of *PDCD1LG2* and *IDO1* mRNA in human lung adenocarcinoma. Each dot represents a lung adenocarcinoma tumor from TCGA (*n* = 510). **C**—**D** RT-qPCR measurement of *CD274* mRNA (**C**) and *PDCD1LG2* mRNA (**D**) induction in lung adenocarcinoma cells stimulated with 5 ng/mL IL-1β for 48 h. **E** Western blots showing PD-L1 protein (*CD274* gene) and IDO1 protein induction in lung adenocarcinoma cells. **F** Schematic showing the experimental set-up for (**G**). **G** Western blot for IDO1 and PD-L1 protein in HCC827 cells at 24 and 48 h after 5 ng/mL IL-1β stimulation and their levels 24 and 48 h after the removal of the stimulation. GAPDH was used as the loading control. Error bars represent ± SD of 3 biological replicates: *P ≤ 0.05, ** ≤ 0.005, *** ≤ 0.0005. mRNA levels were normalized to 18s mRNA and represented as fold change over control treatment

To gain better insights into the biological effects of IL-1β in lung cancer cells, we used the TCGA database to identify genes that are co-expressed with *IDO1* in lung adenocarcinoma patients. We were intrigued to see that the PD-1 ligands PD-L1(*CD274* gene) and PD-L2 (*PDCD1LG2* gene) mRNA were among top significant genes that were positively correlated with *IDO1* mRNA in lung adenocarcinoma tumors (Fig. 5A, B). To determine whether IL-1β is an upstream regulator of these immune checkpoint proteins, we measured PD-L1 and PD-L2 mRNA levels in A549, H1792, H2347, HCC364 and HCC827 in response to IL-1β treatment. Significant upregulation of *CD274* (PD-L1) and *PDCD1LG2* (PD-L2) mRNA (Fig. 5C, D) and PD-L1 protein (Fig. 5E) was detected. Several studies have previously reported that NFκB signaling mediates *PD-L1* mRNA and protein induction in cancer cells [44]. NFκB-p65 signaling however, did not induce *PD-L1* or *PD-L2* mRNA or PD-L1 protein in IL-1β–stimulated HCC827 cells (Supplemental Fig. 2A, B, C). This indicates that IL-1β could be coactivating other pathways or transcription factors to induce PD-L1 and PD-L2 expression in lung adenocarcinoma cells.

We tested whether the transcription factors that have been reported to induce PD-L1 in other cell or cancer types, including CEBPβ, NRF2 [45] and RelB [46] induced PD-L1. However, in IL-1β-stimulated lung adenocarcinoma cells, these transcription factors did not affect the expression of PD-L1 or PD-L2 (Supplemental Fig. 2). STAT signaling is one of the most well-characterized modes of PD-L1 induction in cancer cells [47–49], therefore we tested whether IL-1β induces STAT activation in lung cancer cells. Since IL-1β primarily signals though NFκB pathway [43], we were surprised to see an increase in p-STAT1 and p-STAT3 levels in HCC827 cells treated with IL-1β (Supplemental Fig. 2E).

To assess the duration of elevated protein levels following the cessation of cytokine stimulation, we exposed HCC827 cells to either a control or 5 ng/mL IL-1β treatment for 48 h. Subsequently, the cells were rinsed, and IL-1β-free medium was introduced (Fig. 5F). Both PD-L1 and IDO1 protein levels remain elevated 24 h after IL-1β was removed (Fig. 5G), and only IDO1 levels remained elevated 48 h later. Additionally, we see that the lung cancer cells are sensitive to the species that the cytokine is derived from. The human lung cancer cells require 100 times more of the mouse derived IL-1β to generate an IDO1/PD-L1 response that is seen with the human-derived IL-1β (Supplemental Fig. 3), indicating that further in vivo characterization needs to be performed in syngeneic or humanized mouse models. Together, these data show that IL-1β is indeed an upstream regulator of immune checkpoint proteins IDO1, PD-L1 and PD-L2.

**Fig. 6:**
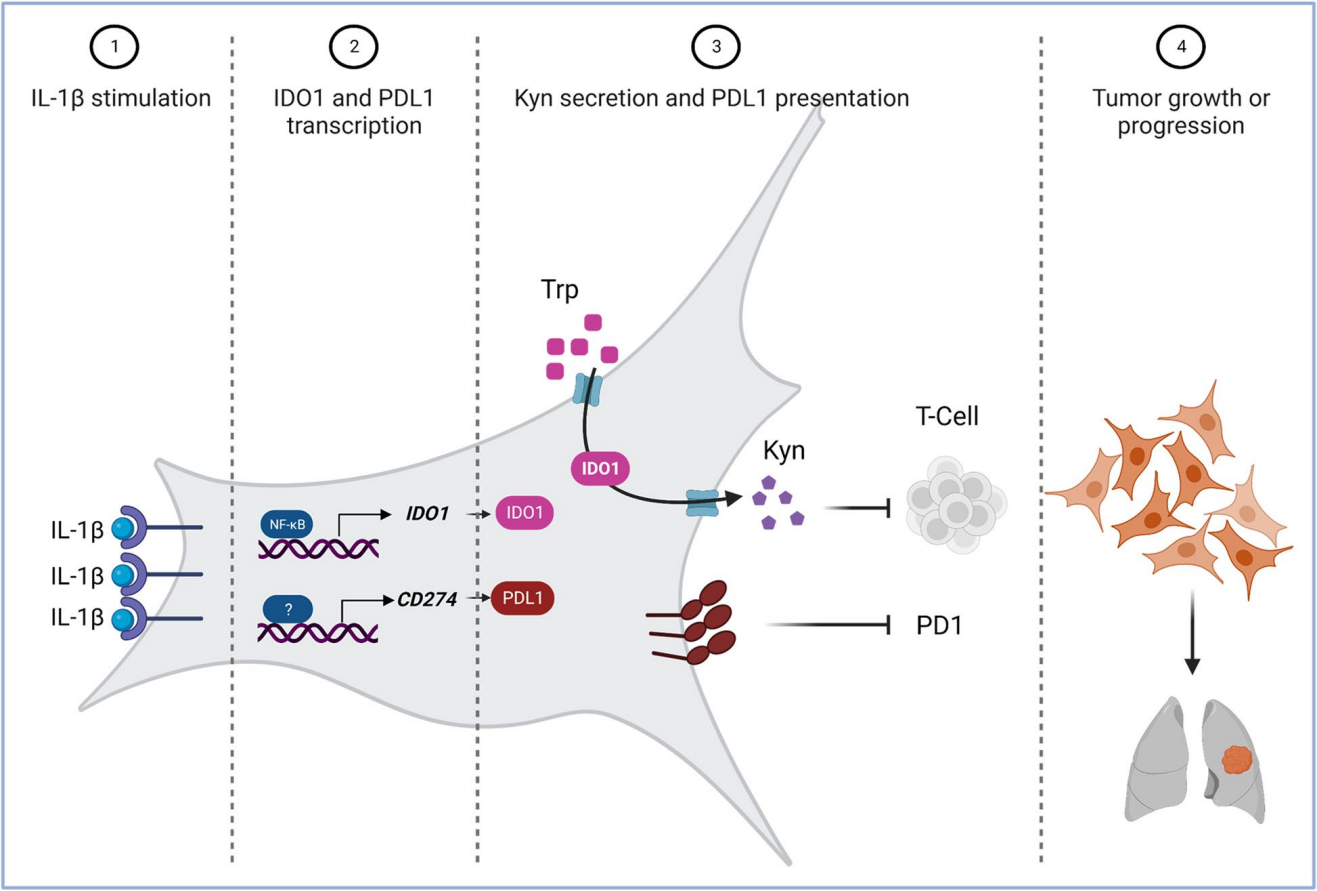
Working model for the regulation of immunomodulators by IL1β in NSCLC

Here, we show that IL-1β-stimulated lung adenocarcinoma cells with various driver mutations respond with an NFκB-mediated upregulation of IDO1. This IDO1 induction is concomitant with an increase in PD-L1 and PD-L2 and together, may provide the lung cancer cells a survival advantage by Kyn-dependent or PD-L1/2-PD-1-mediated inhibition of T-cell activity (Fig. 6).

## Discussion

Inhibiting inflammatory cytokine IL-1β, which is elevated systemically in lung cancer patients [22] and also within lung tumors [20], was effective in reducing lung cancer incidence and mortality [23]. However, the molecular mechanisms underlying this important clinical benefit are not fully understood. Here, we provide novel insights on a mechanism that plausibly underlies this effect: namely the induction of immune checkpoint protein IDO1 by IL-1β in lung adenocarcinoma cells. Importantly, IDO1 induction was observed across a spectrum of lung adenocarcinoma cells lines derived from tumors with varying oncogenic activation. In addition, greater induction of IDO1 mRNA and protein was observed in spheroid models of cell culture, which better recapitulates the 3D cell to cell interactions found in tumors. Similar to previous reports [50], our study shows that IL-1β does not induce IDO1 expression in other normal human cells, despite their ability to respond to interferon gamma with upregulation of IDO1 and to IL-1β with upregulation of CXCL8. These findings identify an important difference between lung cancer and both large and small airway normal lung epithelial cells. Using mouse Lewis lung carcinoma (LLC1) cells, knocking down *IDO1* has been shown to reduce cell growth in vitro [51]. Studies in triple-negative breast cancer cells have also indicated that knockdown of TDO2 induces apoptosis in these cells in vitro, suggesting a cancer cell intrinsic dependency on the Trp catabolizing enzymes for survival [42]. Treating cells with the IDO1 inhibitor epacadostat did not significantly alter cell viability despite a strong IL-1β-mediated induction of IDO1. It is possible that a subset of cells within a spheroid expressing very high IDO1 had lower cell viability with epacadostat incubation. Nevertheless, the IL-1β induction of IDO1 and its product Kyn is likely to reduce the growth of lung cancer cells in vivo due to the ability of Kyn to blunt T-cells activity and allow for immune evasion [9].

Analysis of lung adenocarcinoma patient data set from TCGA revealed that *IDO1* expression was positively correlated with *CD274* and *PDCD1LG2* expression. This is intriguing, because IDO1 catalyzes the conversion of Trp to Kyn, which can suppress T-cell activity and allow tumor cells to escape clearance by the immune system [9], particularly through Kyn-mediated aryl hydrocarbon receptor (AHR) activation [10]. T-cells can take up Kyn in the tumor microenvironment and activate AHR signaling to upregulate the expression of PD-1 on the T-cell surface [10]. PD-1 interacts with PD-L1 or PD-L2 present on cancer cells to limit the cytotoxic T-cell response [10]. For NSCLC without known driver gene mutations, anti-PD-1 or anti-PD-L1 treatment with or without platinum-based chemotherapy has become the first-line strategy, but the overall response rate remains unsatisfactory [52]. Apart from the PD-L1 expression level, various factors, including tumor-infiltrating lymphocytes, tumor mutation burden, neoantigens, and unidentified activated oncogenic pathways, impact patient responses to treatment. Consequently, ongoing studies aim to pinpoint the individuals who would derive the greatest benefit from anti-PD-1/PD-L1 therapies [52]. Considering our discoveries that IL-1β prompts the upregulation of CD274 and PDCD1LG2 mRNA, as well as PD-L1 protein in lung adenocarcinoma cell lines, it is conceivable that heightened tumor or circulating IL-1β might additionally enhance the PD-1/PD-L1 axis’s suppression of cytotoxic T-cells.

Ifnγ [52] and IL-27 also induce the expression of IDO1 and PD-L1 concomitantly, and both act through STAT1 activation to upregulate IDO1 and PD-L1 [52, 53]. STAT signaling is one of the most well-characterized mode of PD-L1 induction in cancer cells [47–49], but IL-1β primarily signals though NFκB pathway [43], therefore, we were surprised to see an increase in p-STAT1 and p-STAT3 levels in HCC827 cells treated with IL-1β. We postulate that the lung cancer cells may be activating an aberrant/non-canonical activation of STAT signaling, leading to induction of PD-L1 and PD-L2—which needs to be further investigated. Although both Ifnγ and IL-27 induce IDO1 and PD-L1 through STAT1 signaling [52, 53], under IL-1β stimulation IDO1 and PD-L1 appear to be regulated through different transcription machinery; however, these immune checkpoint proteins are co-expressed in cancer cells, indicating a cancer cell-intrinsic coupling of IDO1 and PD-L1 expression.

While studies have shown myeloid cell–derived IL-1β to be present in syngeneic models of lung tumors using LLC-1 cells [54], whether IL-1 is present in xenografts and whether these human-derived cancer cells respond to mouse IL-1β has not been studied. Since human lung cancer cells require several fold more of the mouse derived IL-1β to generate an IDO1/PD-L1 response that is seen with the human-derived IL-1β, further, studies need to be done in a syngeneic model such as genetically engineered mouse models (GEMMs), particularly to define whether IL-1 is present in mouse tumors, at what stage, and whether it contributes to tumor progression. The IL-1/IDO1/PD-L1 axis also needs further investigation in vivo*.* IL-1 is a pleiotropic cytokine, so it is important to identify how the different cell types within a tumor respond to the cytokine stimulation and influence tumor progression. Overall, we propose that studies in GEMMs and humanized mice models should be employed to further delineate the mechanism of IL-1β in promoting lung cancer and to determine the efficacy of using IL-1 blockade therapies in combination with other treatments for lung adenocarcinoma. Of course, considerations for the design and implementation of clinical trials combining Canakinumab and immune checkpoint blockade are already ongoing as seen in the CANOPY-N (NCT039648419) [55, 56]. Preclinical studies like ours provide both mechanistic insights and potential biomarkers (IDO1 induction) to evaluate the effectiveness of such approaches.

## Conclusion

Cancer cells have evolved an innate program to respond to different cytokine stimuli (Ifnγ, IL-27, or IL-1) by upregulating both IDO1 and PD-L1 to promote cancer cell survival. Our study has identified IL-1β as an upstream regulator of IDO1/PD-L1 axis of immune suppression in lung adenocarcinoma cells, which is partly through NFκB-p65 activity. These studies indicate that lung adenocarcinoma cells, but not normal lung epithelial cells, are primed to respond to IL-1 signaling by inducing the expression of cytoprotective immune checkpoint proteins, which could promote tumor cell survival through immunosuppression.

### Supplementary Information

**Additional file 1: Table S1.** Primer Sequences.

**Additional file 2: Supplemental Fig. 1.** (A) Heatmap showing the FPKM values of the IL-1 receptors, IL-1 ligands, immune checkpoint genes (from Minna lab RNAseq, dbGaP Study Accession is phs001823.v1.p1 and the fold-change in of CXCL8 and IDO1 mRNA induction in response to 5 ng/ml IL-1β for 48 h, in each of the cell lines. (B) Heatmap showing the FPKM values of TDO2, IDO1, IDO2, CXCL8 and IL-1α mRNA of the cell lines used in this study from the cancer cell line encyclopeAQ11dia (CCLE) database. ﻿

**Additional file 3: Supplemental Fig. 2.** (A-B) HCC827 cells transfected with 40 nM of non-targeting or *RELA*/p65 siRNA for 24 h followed by treatment with vehicle (ctrl) or 5 ng/mL IL-1β for 48 h followed by (A) RT-qPCR for CD274 and PDCD1LG2 mRNA and (B) western blotting for PD-L1 protein. (C) Western blot of PD-L1 in HCC827 cells treated with 5 ng/ml IL-1β ± BAY11-7085 (p65/NFκB inhibitor) for 48 h. (D) HCC827 cells transfected with 40 nM of non-targeting or CEBPβ siRNA for 24 h followed by treatment with vehicle (ctrl) or 5 ng/mL IL-1β for 48 h followed by western blotting for PD-L1. (D) Western blot of p-STAT1 and p-STAT3 in HCC827 cells stimulated with IL-1β for 6 h. GAPDH is loading control.

**Additional file 4: Supplemental Fig. 3.** (A – C) RT-qPCR of *IDO1* (A), *PD-L1* (B) and *PD-L2* (C) mRNA in H1792 and HCC827 cells treated with 1, 10 or 100 ng/mL mouse-derived (mIL-1β) or 1 ng/mL human-derived IL-1β (hIL-1β) for 48 h. Note that *P*-value is represented by comparing all treatments to 1 ng/mL hIL-1β. (D) Western blot of IDO1 and PD-L1 protein in HCC827 cells treated with 5 ng/mL IL-1β. GAPDH is loading control. Error bars represent ± SD of 3 biological replicates: *P ≤ 0.05, ** ≤ 0.005, *** ≤ 0.0005. mRNA levels were normalized to 18s mRNA and represented as fold change over control treatment.

## Data Availability

All data and materials used are available within the manuscript.

## Abbreviations

AHR: Aryl hydrocarbon receptor
ALK: Anaplastic lymphoma kinase
BRAF: V-raf murine sarcoma viral oncogene homolog B
CANTOS: Canakinumab Anti-inflammatory Thrombosis Outcomes Study
CCLE: Cancer Cell Line Encyclopedia
CDK4: Cyclin-dependent kinase 4
EGFR: Epidermal growth factor receptor
HRMS: High-resolution mass spectrometer
hTERT: Human telomerase reverse transcriptase
IDO1: Indoleamine 2, 3-dioxygenase-1
Ifnγ: Interferon gamma
IL-1β: Interleukin-1 beta
KRAS: Kirsten rat sarcoma viral oncogene homolog
Kyn: Kynurenine
LCMS: Liquid chromatography mass spectrometry
LKB1: Liver kinase B1
NSCLC: Non-small cell lung cancer
PD-L1: Programmed death ligand 1
PD-L2: Programmed death ligand 2
TCGA: The Cancer Genome Atlas
TDO2: Tryptophan 2,3-dioxygenase
TIC: Total ion count
Trp: Tryptophan
