# Supplementary material for: IL-1β mediates the induction of immune checkpoint regulators IDO1 and PD-L1 in lung adenocarcinoma cells
Source: Cell Commun Signal. 2023 Nov 20;21:331. doi: 10.1186/s12964-023-01348-1 (PMC10658741; doi:10.1186/s12964-023-01348-1)
Supplement: Supplementary file 2 — Additional file 1: Table S1. Primer Sequences. [file 12964_2023_1348_MOESM1_ESM.pdf]

Table S1. Primer Sequences

Gene	Forward	Reverse
IDO1	GGGAAGCTTATGACGCCTGT	CTGGCTTGCAGGAATCAGGA
TDO2	GGAGGCATGGCTGGAAAGAA	TCTTCCAGGCCTCTGGTGAT
PD-L1/ CD274	CCTCTGGCACATCCTCCAAA	TCAGTGCTACACCAAGGCAT
PD-L2/ PDCD1LG2	CATCCCCACACCGTGAAAGA	CCTTCGTCCCTCACTTGGAC
RelA/p65	TGAACCAGGGCATACCTGTG	CCCCTGTCACTAGGCGAGTT
IL-8	ACACTGCGCCAACACAGAAAT	AACTTCTCCACAACCCTCTGC
18s	ACCGCAGCTAGGAATAATGGA	GCCTCAGTTCCGAAAAACA
