# Supplementary figures and images for: IL-1β mediates the induction of immune checkpoint regulators IDO1 and PD-L1 in lung adenocarcinoma cells
Source: Cell Commun Signal. 2023 Nov 20;21:331. doi: 10.1186/s12964-023-01348-1 (PMC10658741; doi:10.1186/s12964-023-01348-1)

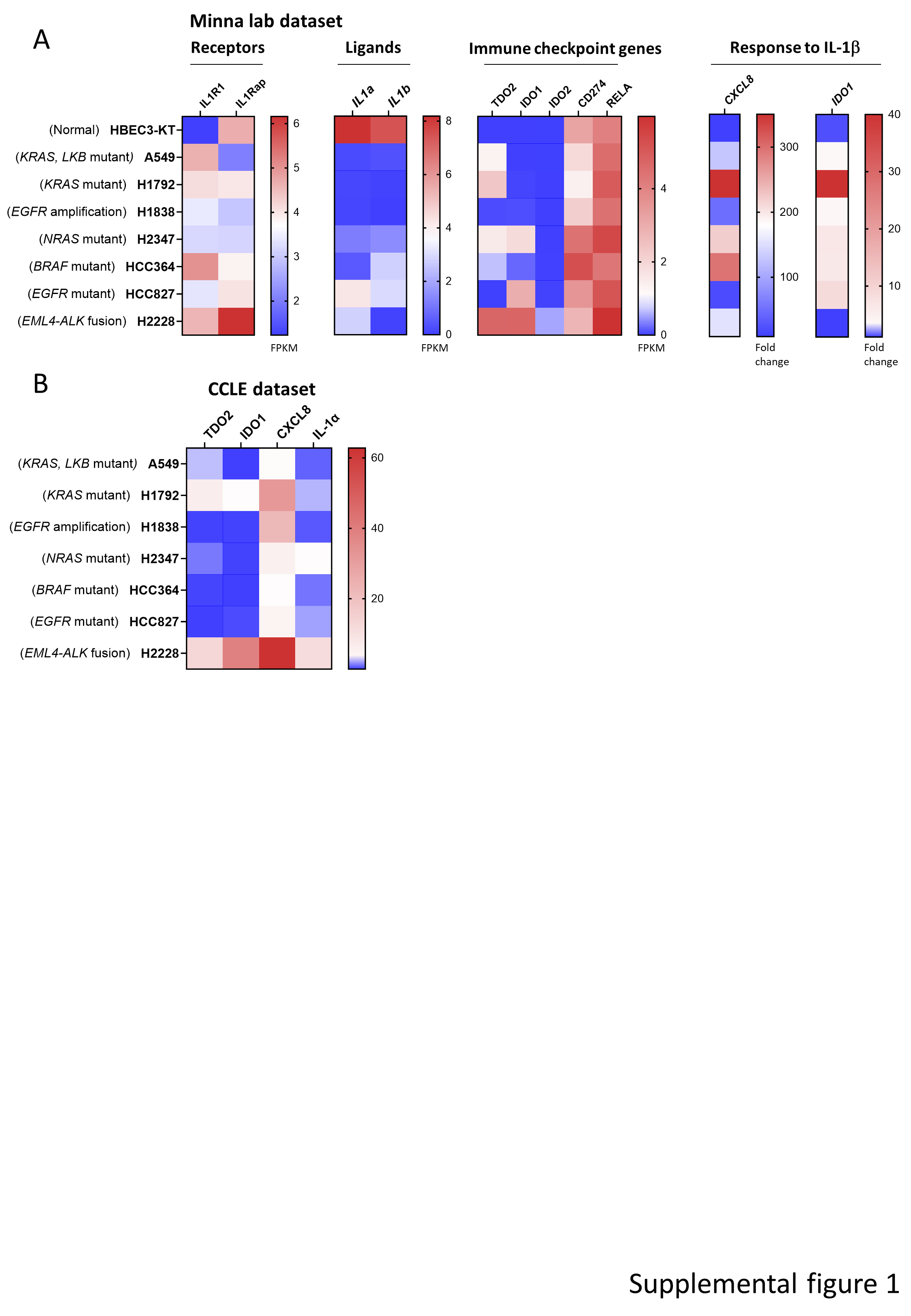

Supplement: Supplementary file 3 — Additional file 2: Supplemental Fig. 1. (A) Heatmap showing the FPKM values of the IL-1 receptors, IL-1 ligands, immune checkpoint genes (from Minna lab RNAseq, dbGaP Study Accession is phs001823.v1.p1 and the fold-change in of CXCL8 and IDO1 mRNA induction in response to 5 ng/ml IL-1β for 48 h, in each of the cell lines. (B) Heatmap showing the FPKM values of TDO2, IDO1, IDO2, CXCL8 and IL-1α mRNA of the cell lines used in this study from the cancer cell line encyclopeAQ11dia (CCLE) database. ﻿ [file 12964_2023_1348_MOESM2_ESM.tif]

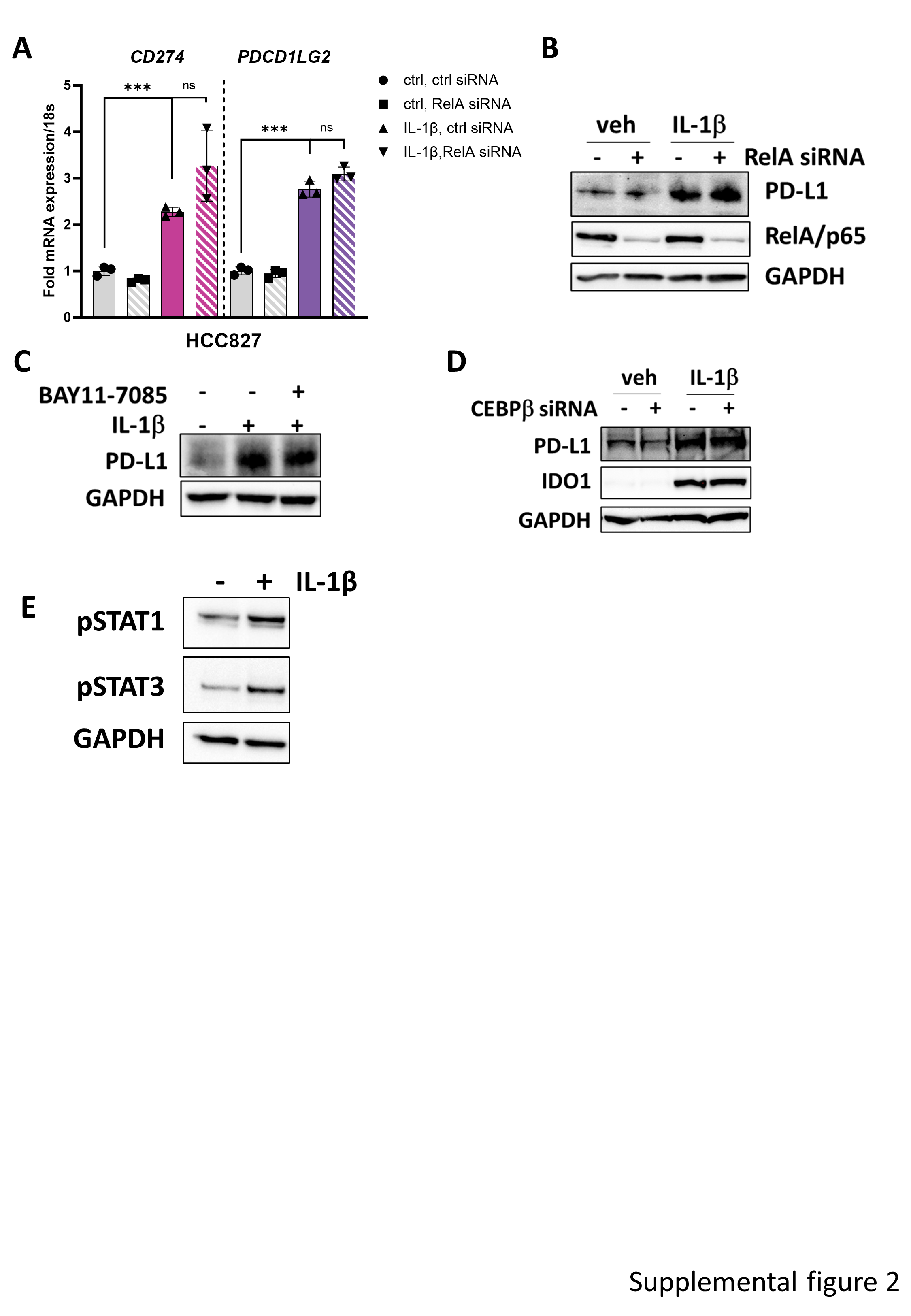

Supplement: Supplementary file 4 — Additional file 3: Supplemental Fig. 2. (A-B) HCC827 cells transfected with 40 nM of non-targeting or RELA/p65 siRNA for 24 h followed by treatment with vehicle (ctrl) or 5 ng/mL IL-1β for 48 h followed by (A) RT-qPCR for CD274 and PDCD1LG2 mRNA and (B) western blotting for PD-L1 protein. (C) Western blot of PD-L1 in HCC827 cells treated with 5 ng/ml IL-1β ± BAY11-7085 (p65/NFκB inhibitor) for 48 h. (D) HCC827 cells transfected with 40 nM of non-targeting or CEBPβ siRNA for 24 h followed by treatment with vehicle (ctrl) or 5 ng/mL IL-1β for 48 h followed by western blotting for PD-L1. (D) Western blot of p-STAT1 and p-STAT3 in HCC827 cells stimulated with IL-1β for 6 h. GAPDH is loading control. [file 12964_2023_1348_MOESM3_ESM.tif]

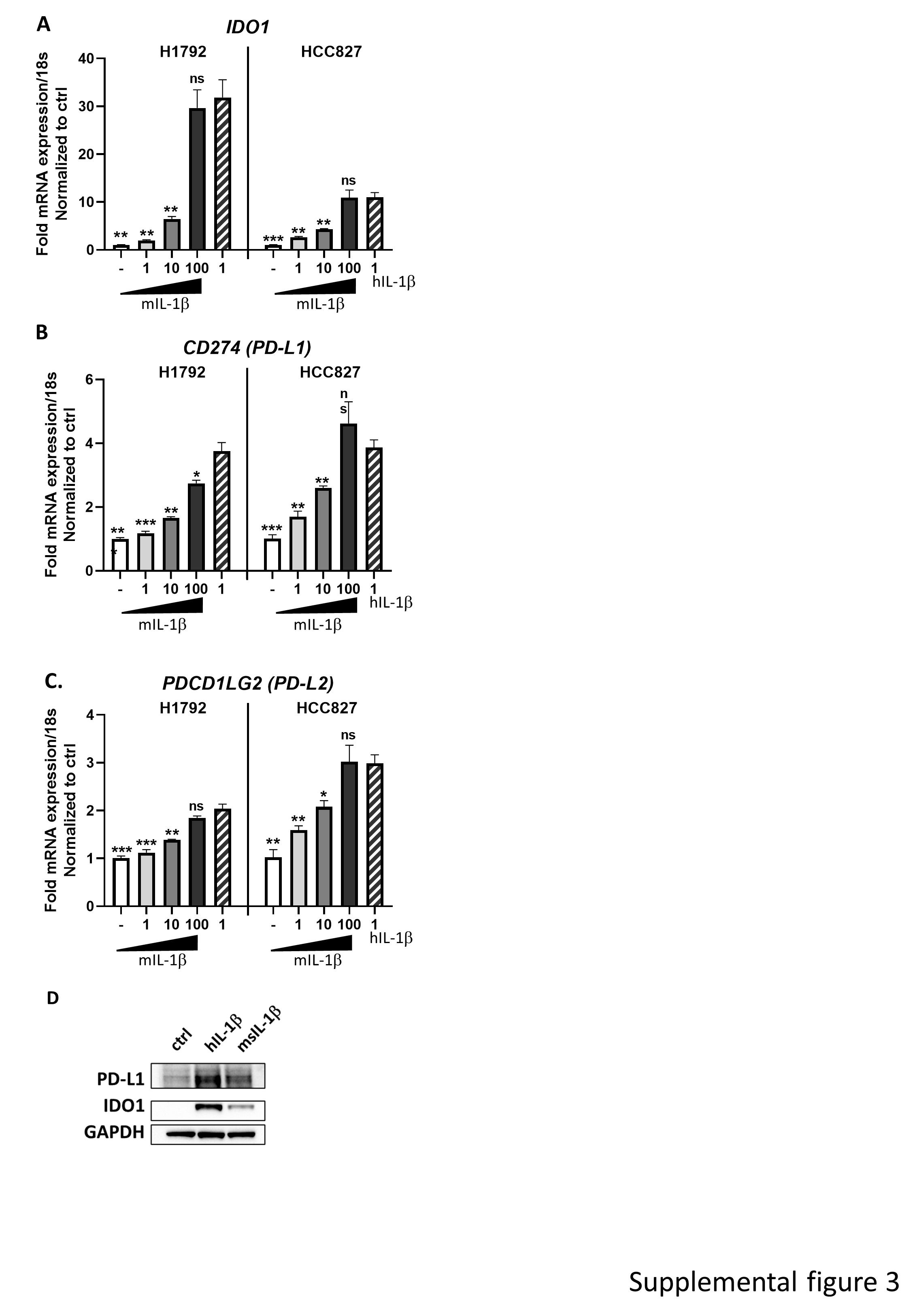

Supplement: Supplementary file 5 — Additional file 4: Supplemental Fig. 3. (A – C) RT-qPCR of IDO1 (A), PD-L1 (B) and PD-L2 (C) mRNA in H1792 and HCC827 cells treated with 1, 10 or 100 ng/mL mouse-derived (mIL-1β) or 1 ng/mL human-derived IL-1β (hIL-1β) for 48 h. Note that P-value is represented by comparing all treatments to 1 ng/mL hIL-1β. (D) Western blot of IDO1 and PD-L1 protein in HCC827 cells treated with 5 ng/mL IL-1β. GAPDH is loading control. Error bars represent ± SD of 3 biological replicates: *P ≤ 0.05, ** ≤ 0.005, *** ≤ 0.0005. mRNA levels were normalized to 18s mRNA and represented as fold change over control treatment. [file 12964_2023_1348_MOESM4_ESM.tif]
